# Quantum sensing with optically accessible spin defects in van der Waals layered materials

**DOI:** 10.1038/s41377-024-01630-y

**Published:** 2024-11-05

**Authors:** Hong-Hua Fang, Xiao-Jie Wang, Xavier Marie, Hong-Bo Sun

**Affiliations:** 1 State Key Laboratory of Precision Measurement Technology and Instruments, Department of Precision Instrument, Tsinghua University, 100084 Beijing, China; 2 Université de Toulouse, INSA-CNRS-UPS, LPCNO, 135 Avenue Rangueil, 31077 Toulouse, France; 3 Institut Universitaire de France, 75231, Paris, France

**Keywords:** Optics and photonics, Optical physics

## Abstract

Quantum sensing has emerged as a powerful technique to detect and measure physical and chemical parameters with exceptional precision. One of the methods is to use optically active spin defects within solid-state materials. These defects act as sensors and have made significant progress in recent years, particularly in the realm of two-dimensional (2D) spin defects. In this article, we focus on the latest trends in quantum sensing that use spin defects in van der Waals (vdW) materials. We discuss the benefits of combining optically addressable spin defects with 2D vdW materials while highlighting the challenges and opportunities to use these defects. To make quantum sensing practical and applicable, the article identifies some areas worth further exploration. These include identifying spin defects with properties suitable for quantum sensing, generating quantum defects on demand with control of their spatial localization, understanding the impact of layer thickness and interface on quantum sensing, and integrating spin defects with photonic structures for new functionalities and higher emission rates. The article explores the potential applications of quantum sensing in several fields, such as superconductivity, ferromagnetism, 2D nanoelectronics, and biology. For instance, combining nanoscale microfluidic technology with nanopore and quantum sensing may lead to a new platform for DNA sequencing. As materials technology continues to evolve, and with the advancement of defect engineering techniques, 2D spin defects are expected to play a vital role in quantum sensing.

## Graphical abstract

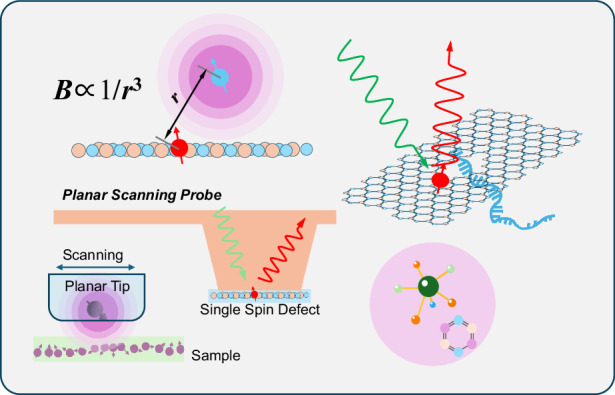

## Introduction

Quantum-enhanced sensing techniques have significantly improved our ability to measure physical properties with ultra-precision, including electromagnetic fields^1,2^, temperature^3–6^, and frequency^7,8^. One such method involves using optically active spin defects within solid-state materials as sensors^9–16^. These sensors comprise atom-like systems within a solid material, giving them well-defined energy levels that can be optically manipulated and measured with high precision^14,17^. By detecting changes in the energy level spacing in response to environmental changes, these sensors can uncover small modifications in their surroundings. Common sensors use spin defects found in wide-bandgap semiconductors like diamond and silicon carbide^18,19^. These defects are naturally present in the material, arising from atomic-level imperfections where the electron spins within the atoms do not align perfectly, creating small magnetic dipoles. Using laser techniques, the spin state of these defects can be initialized and precisely controlled. They are also optically detectable, providing a novel approach to investigating quantum phenomena at the atomic scale^20^.

These optically active spin defects, with their remarkable quantum coherence^14,21^, single-spin addressability, and ultra-high field sensitivity, play a pivotal role in advancing quantum technologies^22,23^, biomedical sciences^24–26^, and material science^13^, among other fields. Among these defects, the negatively charged nitrogen-vacancy (NV^-^) center is a leading candidate for quantum sensing applications^21,27–30^. Its unique properties, such as its long-lived spin coherence, optical spin initialization, and measurement capacity, make it particularly appealing^31^. Additionally, its proximity to individual nuclear spins enables precise interactions and measurements^9,10,27,32,33^. The NV center’s optical and spin properties were first discovered in the 1970s^34,35^, but it wasn’t until a decade later that the optical detection of magnetic resonance (ODMR) was achieved^36,37^. In the early 1990s, ODMR experiments on individual NV centers were conducted at room temperature^38^, shortly after the emergence of single-molecule spectroscopy in condensed matter^39,40^. In 2008, several research groups independently advanced the field of diamond-based magnetometry^9,10,32,33^. Taylor et al. and Degen proposed using diamond nanocrystals as magnetic field sensors^32,33^. Around the same time, Maze et al. and Balasubramanian et al. demonstrated using a single NV center in diamond as a scanning probe magnetometer^9,10^.

**Fig. 1:**
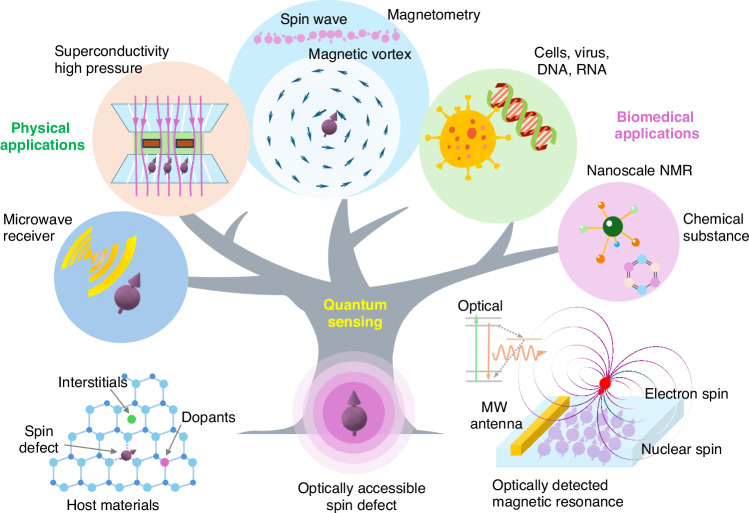
An overview of quantum sensing with an optically accessible spin center. Quantum sensing with optically accessible spin centers involves using paramagnetic defects in solids, such as nitrogen-vacancy (NV) centers in diamonds. Optically detected magnetic resonance (ODMR) is used to read the spin of solid-state color centers, which enables the creation of spin-based quantum sensors for measuring magnetic fields, electric fields, and temperature with high sensitivity. These sensors have broad applications in areas like microwave detection, superconductivity, and magnetic materials. Additionally, spin defects can be utilized in nanoscale nuclear magnetic resonance (NMR) spectroscopy. Ultrasensitive magnetometers can be used to perform nanoscale NMR. This technology has been used for detecting viruses, single proteins, and single protons

In the decade that followed, single-spin and ensemble-averaged NV-diamond magnetometers have undergone significant advancements. Figure 1 shows an overview of quantum sensing with optically accessible spin centers, which can measure magnetic fields, electric fields, temperature, etc., and eventually, they have versatile applications in fields, such as condensed matter physics^41,42^, and radio receiver^1,43^, and biology^44,45^, like protein and virus detections. In condensed matter physics, it has been applied for the measurement of magnetic field textures in correlated electron systems, advancing our understanding of materials’ behavior under extreme conditions^46–48^. Its application in superconductivity research allows for exploring the quantum properties of superconducting materials at extremely high pressure^49^. Moreover, integrating NV-based quantum sensing with CMOS technology drives the development of compact and scalable platforms^50^. Spin defects in biology are capable of being used as nanoscale nuclear magnetic resonance spectroscopy to perform ultrasensitive magnetometers, which can detect viruses, proteins, and protons. This technology has revolutionized medical diagnostics and treatment by monitoring and imaging biomagnetism. It is particularly effective in detecting the RNA of viruses for diagnostics^11,44,51,52^.

Following the success of diamond color centers in quantum sensing applications, researchers have also been exploring new color center systems. Some of these systems include Germanium vacancy (GeV) and Silicon-vacancy (SiV) in diamond^53,54^, spin defect in other wide-bandgap semiconductors such as silicon vacancy centers in SiC^55,56^, divacancy centers in SiC^57^, and recently reported single color centers in GaN and AlN^58,59^. Among the explored color centers, some have demonstrated exceptional optical properties, including bright zero-phonon lines, large Debye-Waller factors, and lifetime-limited line widths. These properties make them promising candidates for use in photonic quantum technologies as spin-photon interfaces. Some of these color centers have a controllable spin state and a coherence time significantly longer than the time required to perform basic operations on the state, which makes them well-suited for applications in quantum sensing or quantum computing.

In parallel, there has been a surge of interest in van der Waals layered materials, attracting attention due to their intriguing physics, distinctive electronic properties, and unique structural characteristics^60–64^. One of the most fascinating aspects of van der Waals materials is the ability to isolate monolayers. This ability not only opens up new possibilities for manipulating and controlling these effects but also allows for the engineering of spin centers in layered materials in close proximity to target materials^60,65^. This is extremely important for magnetic field sensing. It can improve spatial resolution and sensitivity, as the magnetic field strength **B** at a distance **r** from the dipole is inversely proportional to the cube of the distance: **B** ∝ 1**/r**^3,66^.

**Fig. 2:**
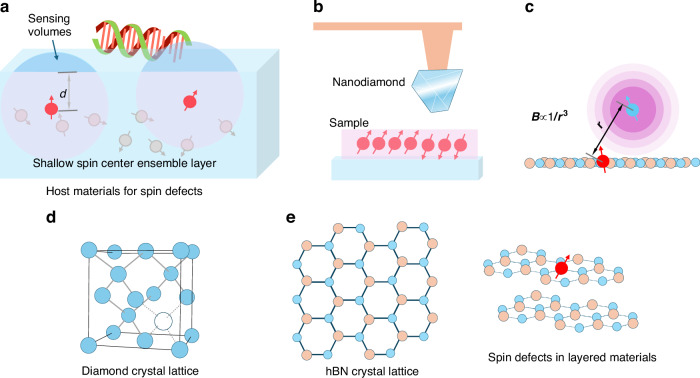
Magnetic field sensing depends on the distance between the sample and the detecting spin. **a** NV centers in diamonds that are aligned with the external magnetic field have sensing volumes that depend on their depth relative to the diamond’s surface. NV centers that are located closer to the diamond’s surface are more responsive to magnetic fields that are induced by the Larmor precession of nuclei from the sample outside of the diamond. **b** The dipolar fields originating from spins within a sample decrease exponentially with distance. Only spins that are located within a certain distance from a spin detector, like a nitrogen-vacancy center, will contribute to the measurable signal. The distance between the spin center and the surface of the sample material determines the effective spatial resolution. **c** Bringing a magnetometer into proximity to a field source provides a significant detection sensitivity advantage as the magnetic field strength decreases with distance according to ~1/*r*^3^. **d** Representation of the 3D crystal structure of diamond materials, showcasing their 3D atomic arrangement. **e** A monolayer of two-dimensional materials, which have an atomic thickness, benefits from the proximity effect, allowing for enhanced physical and electrical properties due to their proximity to other materials or fields

As the distance between the sensor and the magnetic source decreases, the detected magnetic field strength increases dramatically^66^. Consequently, by operating at very short distances, sensors can achieve higher resolution and greater sensitivity, as they can detect the subtle changes in the magnetic field caused by small changes in proximity to the source^67^. Figure 2 illustrates the importance of the distance in magnetic field sensing applications. It is crucial to induce an ensemble of NV centers layer near the surface layer for wide-field magnetic imaging (Fig. 2a). The effective spatial resolution in a scanning magnetic field microscope with a single-spin sensor depends on the distance between the spin center and the surface of the sample material (Fig. 2b). This concept has been a pivotal force in developing near-surface nitrogen-vacancy (NV) centers over the past decade. Various techniques such as nitrogen delta-doping and the creation of NV centers within nanoscale diamond crystals have been developed to achieve this^68,69^. From this perspective, van der Waals layered materials have shown clear benefits compared to conventional 3D semiconductors (Fig. 2c–e). They allow spin sensors to be placed mere atoms away from the target.

Additionally, due to their self-passivated, dangling-bond-free surfaces, two-dimensional (2D) layered materials can readily be integrated with non-2D materials through van der Waals (vdW) interactions^70^. This capability allows for routine and seamless assembly of vdW materials into multifunctional heterostructures, which is promising for in situ quantum sensing. Such advancements could lead to widespread applications across various devices and scenarios, providing a promising platform for creating novel quantum sensors with significantly enhanced capabilities.

In this Perspective, we discuss current trends in quantum sensing utilizing spin defects within van der Waals (vdW) materials. Specifically, we highlight the potential benefits of combining two-dimensional (2D) vdW materials with optically addressable spin defects. We also explore the latest developments in quantum microscopes using 2D spin defects, the techniques used to create these defects, and the challenges and opportunities associated with this type of quantum sensing. Our main objective is to identify the most promising 2D quantum sensors and highlight the critical research needs in this field. The potential of new opportunities for their application is discussed. By focusing on these, we aim to promote a more targeted and productive research methodology.

## Optically active defects in ultrathin van der Waals layered materials

### Quantum defect in van der Waals layered materials

**Fig. 3:**
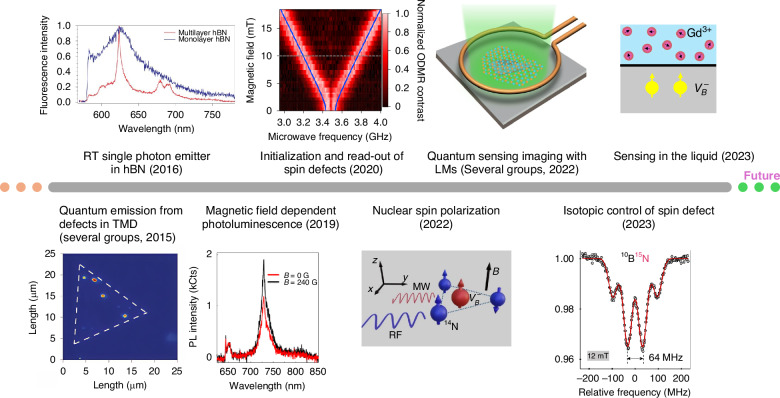
Some representative works in the quantum defects within layered quantum materials and their development towards quantum sensing applications. Given the rapid development of this field, we can only list part of them. For example, some of the research topics related to quantum emission from defects in transition metal dichalcogenides (TMDs)^71–74^, room temperature single photon emitters in hBN^75^, magnetic field-dependent photoluminescence^102^, initialization and read-out of spin defects^105^, nuclear spin polarization^158^, quantum sensing imaging with layered materials (LMs)^147^, isotopic control of spin defects^120^, and sensing in liquids^229^. Figures adapted and reprinted with permission^72,75,102,105,120,147,158,229^. Copyright by Springer Nature^72,75,102,105,147,158^, American Physical Society^120^, and American Chemical Society^229^

In 2015, several groups independently observed quantum emissions from monolayer and bilayer transition metal dichalcogenides (TMDs)^71–74^. Soon after, similar findings were reported in multilayer hexagonal boron nitride (hBN)^75^. These discoveries have sparked a profound interest in the quantum defects in 2D materials, creating new opportunities for quantum photonics and beyond^76^. Various quantum defects in layered quantum materials and their development towards quantum sensing applications are shown in Fig. 3.

Owing to their low dimensionality, 2D materials showcase superior optical properties for spin-photon interfaces. Firstly, these semiconductors are ultrathin, only a few atoms thick, which inherently confers structural “openness” and high light transparency^77^. This thinness obviates the traditional challenges associated with photon extraction, as light can readily traverse the material. Secondly, defects remain largely unscreened in ultrathin layers, maintaining a charge state that does not reach equilibrium with the surrounding material^78,79^. This leads to deeper and more tightly confined defect states, which are distinctly separated from the band edges. This clear separation between the ground and excited states is advantageous for establishing a two-level quantum system characterized by its presence in either the ground or excited state. Thirdly, the minimized screening in 2D materials results in reduced practical defect Bohr radius, elevated radiative rates, and higher oscillator strengths for optical transitions^80^. Additionally, the Franck-Condon (FC) shift associated with defects in 2D TMDs is notably small^81^, further improving the radiative efficiency of transitions within these defect states. Collectively, these properties make 2D materials particularly promising for spin-photon interfaces^82^.

Quantum emission in TMDs is primarily caused by the confinement of excitons linked to strain-induced profiles^83,84^. Recent studies have shown that irregularities in the crystal lattice due to impurities, vacancies, or structural imperfections, also known as point defects, can cause single-photon emission^83,85,86^. These defects introduce localized states within the bandgap of TMDs, which can host electrons and holes that form bound excitons. It’s worth noting that the quantum emission from defects in TMDs usually occurs at cryogenic temperatures^73^. This is because thermal energy can cause confined excitons to delocalize or defect states to be occupied at higher temperatures, making quantum emissions unstable.

Quantum defects in hBN are notable for their ability to remain stable with quantum emission even at temperatures much higher than ambient^71,87–89^. This stability can be attributed to the unique properties of hBN as an insulator with a substantial bandgap of ~6 eV. Although hBN is often referred to as white graphene, it has similarities to the diamond in its electronic structure and optical properties^90^. Moreover, hBN materials are highly biocompatible, exhibit minimal cytotoxicity, and possess exceptional thermal and chemical stability, making them ideal for various applications, particularly in biomedical sensors. Over the years, hBN has emerged as one of the most extensively investigated 2D materials^91^. Defects within hBN, which have electronic levels that fall within the bandgap, are identified as possible sources of single-photon emitters (SPEs). These defects’ zero-phonon line (ZPL) energies usually range from around 1.6 to 2.9 eV, which falls within the near-infrared to the visible spectrum^92–99^. Quantum emitters within hBN exhibit considerable potential, demonstrating high photostability, remarkable brightness, a significant Debye-Waller factor, and superior polarization contrast, which are highly desired for quantum photonics^100^.

### Optically addressable spin defects

Optically addressable spin defects have a crucial role in bridging photons and electron spin states, forming the basis for developing quantum sensing technologies^101^. Exarhos et al. documented the observation of strongly anisotropic photoluminescence patterns as a function of the applied magnetic field for selected quantum emitters within hBN^102^. These findings suggest the presence of optically addressable spin defects within hBN. More recently, these defects have been identified in layered materials, with a particular focus on hBN^3,101,103–108^. However, the types of defects that have demonstrated ODMR properties are still limited^109^. The energy levels of most reported paramagnetic defects are still poorly understood and require further investigation. Several timely reviews have introduced the photophysical properties of quantum defects in two-dimensional materials^108^. In this text, we will briefly introduce some of the investigated and newly emerging spin defects.

### Negatively charged boron vacancy

**Fig. 4:**
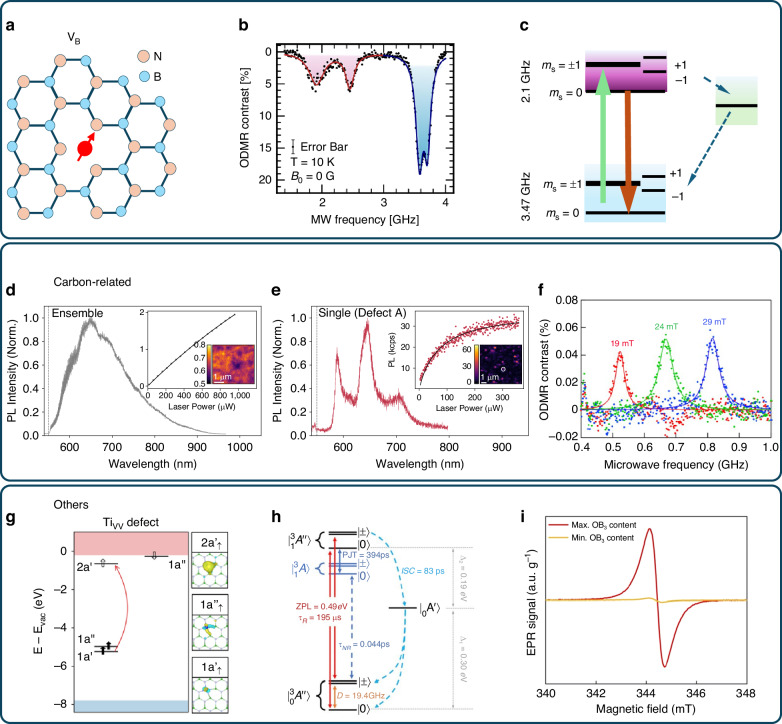
Representation of various spin defects. **a** Depiction of the structural configuration of a boron vacancy (${{\rm{V}}}_{B}^{-}$) defect in hBN. **b** Zero-field ODMR spectrum of ${{\rm{V}}}_{B}^{-}$, showing resonance dips corresponding to spin transitions within the electronic state (in red), and the ground state (blue)^115^. **c** Simplified energy level structure of the ${{\rm{V}}}_{B}^{-}$ center in hBN^115,120^. **d** Photoluminescence (PL) spectrum of an ensemble of carbon-related spin defects within hBN^109^. **e** PL spectrum of an individual carbon-related spin defect. **f** ODMR of a carbon-related spin defect^137^. **g** Calculated defect levels of Ti_VV_ defect in hBN^140^. **h** Energy structure of the Ti_VV_ defect and their recombination rates. **i** Room temperature X-band EPR spectra of paramagnetic OB_3_ in boron oxynitride (BNO) samples^143^. Figures adapted and reprinted with permission from refs. ^109,115,137,140,143^. Copyright by Springer Nature^109,115,137,140^ and American Chemical Society^143^

The negatively charged boron vacancy (${{\rm{V}}}_{B}^{-}$) is a spin defect that has garnered the most attention in this current research in hBN^105,110–118^. This defect is characterized by the absence of a boron atom and the presence of an additional electron in the hBN crystal, as shown in Fig. 4a. In 2018, Toledo et al. reported on the effects of neutron irradiation on hBN, discovering a new paramagnetic defect that imparts a pink color and near-infrared luminescence^119^. This defect involves doubly occupied nitrogen vacancies and shows a near-infrared photoluminescence band centered at 820 nm. Later, in 2020, Gottscholl and colleagues demonstrated the optical initialization and readout of an ensemble of spins in hBN^105^. They utilized electron paramagnetic resonance (EPR) spectroscopy and ODMR measurements to observe a triplet ground state with a zero-field splitting (ZFS) of ~3.5 GHz and an isotropic Landé factor of *g* = 2.000. By analyzing the angular dependence and nitrogen hyperfine structure, they identified it as a ${{\rm{V}}}_{B}^{-}$. The scientific community now has a relatively clear understanding of the energy level structure of the ${{\rm{V}}}_{B}^{-}$ center^115,120^, as illustrated in Fig. 4c. The ${{\rm{V}}}_{B}^{-}$ center is characterized by a ground state that is a spin triplet (*S* = 1), which exhibits a zero-field splitting (D_g_) of roughly 3.47 GHz between the electron spin sublevels m_s_ = 0 and m_s_ = ±1. The m_s_ notation denotes the electron spin projection along the c-axis of the crystal. Optical excitation can elevate the ${{\rm{V}}}_{B}^{-}$ center to an excited state, which remains a spin triplet with a zero-field splitting parameter D_e_ of ~2.1 GHz^120–122^. The relaxation of this excited state back to the ground state can take place either through the emission of a broad near-infrared photoluminescence (PL) signal or undergo nonradiative processes that involve transitions to metastable singlet states^120,123,124^.

### Carbon-related defects

Carbon-related defects are a significant category of paramagnetic defects in hBN that may be introduced during growth, exfoliation, or thermal annealing^94,105,125–129^. The study of electron spin resonance (ESR) in carbon-doped hBN dates back to as early as 1972^130,131^. These defects are considered a source of zero-phonon line (ZPL) emitters in the 1.6–2.2 eV range^132,133^, which is associated with single-photon emission. Recent theoretical investigations have shown that carbon atoms within hBN can create localized states and spin moments^126,132,134^. Wu and colleagues have investigated theoretically several types of defects in hBN monolayers, examining their charge transition levels, stable spin states, and optical properties^135^. They have identified the carbon substitution adjacent to a nitrogen-vacancy (C_B_V_N_) defect as a prime candidate for applications in quantum bits and emitters. Chen has employed first-principles calculations and group theory to study the electronic structures of C_B_V_N_ centers^136^, which are composed of a nitrogen-vacancy and a carbon atom in various charge states. These investigations indicate that the neutral C_B_V_N_ center, with a triplet ground state and two spin-conserved transitions, is stable in n-type hBN.

In recent experiments, Mendelson and colleagues have provided compelling evidence that visible single-photon emissions (SPEs) are associated with carbon-related defects^137^. Through precise impurity incorporation techniques and ion implantation, they have achieved direct observation of these emissions, confirming that SPEs in the visible spectral range are exclusively induced by carbon implantation. Computational investigations of the most fundamental carbon-containing defects indicate that the negatively charged V_B_C_N_- defect is a plausible candidate for SPEs. These studies also indicate that this defect is sensitive to out-of-plane deformations and its local environment. Chejanovsky et al. discovered a collection of isolated optical emitters in hBN that manifest ODMR^107^. The magnetic resonance spectrum obtained is narrow and inhomogeneously broadened, distinct from the spectra of in-plane defects that are previously known. They measured a hyperfine coupling of around 10 MHz, and its angular dependence suggests the presence of an unpaired, out-of-plane delocalized π-orbital electron, which is likely derived from a carbon substitutional impurity. Stern et al. described room temperature ODMR observations from individual defects in hBN^109^, which were attributed to carbon impurities. They measured the photoluminescence (PL) spectrum of an ensemble of carbon-related spin defects and an individual carbon-related spin defect (Fig. 4d, e). They found that the ODMR signal contrast for these single defects can exceed 30%, which is ~100 times greater than the 0.4% ensemble average contrast. Guo et al. reported a novel type of defect displaying ODMR at room temperature. The ODMR positive peak exhibits a contrast of 0.8% and a linewidth of 37 MHz^138^. According to ab initio calculations, this defect could be a complex involving both carbon and oxygen dopants.

It is worth noting that although there is now considerable evidence that carbon-related defects are a significant source of spin defects, the variety of carbon-related defects that have been identified is extensive, such as carbon monomers, dimers, trimers, and larger carbon clusters, as well as complexes of carbon with vacancies, antisites^128^. No single study to date has provided a comprehensive and detailed understanding of their microscopic structure and energy level structure, similar to that achieved for the valence bond (V_B_) model. This represents one of the key challenges that subsequent research needs to address.

### Other type defects

The exploration of spin-related defects in two-dimensional materials is a developing field that has yet to be fully explored^139^. Apart from V_B_ and carbon-related spin defects, the scientific community is actively pursuing the discovery of new spin defects, which at this stage, is mostly theoretical and waiting for experimental validation and a deeper understanding. In their 2020 work, Smart and colleagues developed a theoretical framework that enables the design of quantum defects within two-dimensional (2D) systems, considering both static and dynamic properties to facilitate the discovery of spin qubits^140^. They successfully identified several spin qubits, including Ti_VV_ and Mo_VV_, within hBN (Fig. 4g, h). These transition metal complex vacancy defects are characterized by a stable spin-triplet ground state and possess a substantial zero-field splitting. According to Bhang and colleagues, the X_N_Y_i_ dimer defects (where X and Y can be C, N, P, or Si) constitute a novel class of stable C_3v_ spin-triplet defects^141^. Notably, the zero-phonon line for these spin-triplet X_N_Y_i_ defects occurs within the visible spectrum, ranging from 500 to 800 nm. More recently, Li et al. have uncovered the negatively charged oxygen vacancy defect, V_B_O_N_, as the source of a novel electron paramagnetic resonance (EPR) center^142^. The negatively charged defect creates both occupied and unoccupied states within the gap that is anticipated to be optically active. The zero-field splitting in the S = 1 ground state is at around 2 GHz, which is smaller than that observed in the V_B_ defect. An intersystem crossing (ISC) transition, labeled as (Γ_0,1_), is identified between spin sublevel m_s_ = 0 in triplet and singlet states. This transition allows for the manipulation of the qubit states using microwave pulses, offering potential quantum sensing applications in these systems. Mistry et al. reported paramagnetic properties from isolated OB_3_ states (Fig. 4i)^143^. The spin properties of these defects, however, have not yet been fully experimentally validated, thus requiring further investigation. In summary, two-dimensional materials, which have various spin defects^80,94,126,139,141,144^, offer a robust and promising foundation for advancements in quantum sensing.

## Quantum sensing applications

In 2019, Exarhos et al. evidenced magnetic field-dependent photoluminescence in hBN^102^. The study of spin defects within layered structures has rendered them valuable for the detection of magnetic fields, temperature, and strain, especially for the investigation of novel 2D materials, such as ferromagnetic materials. These layered systems are characterized by their flexibility and modularity, which facilitates their seamless integration with a variety of distinct two-dimensional materials^62^, thereby enhancing their applicability in the fabrication of diverse and customized structures, expanding the potential of these materials in the field of sensors and quantum technology. A study conducted by Gottscholl et al. in 2020 explored spin-dependent processes in hBN^105^, with a focus on the initialization and read-out of intrinsic spin defects. Through the use of electron paramagnetic resonance techniques and photoluminescence spectroscopy, they identified fluorescence lines associated with the negatively charged boron vacancy. The study revealed that this defect exhibits ODMR at room temperature. Additionally, they showed that these centers can be used as atomic-scale sensors capable of detecting temperature changes, magnetic fields, and external pressure^3^.

A standard spin Hamiltonian can be expressed as^145^

1 $$H={H}_{e}+{H}_{n}+{H}_{{en}}$$

where ${H}_{e}$, ${H}_{n}$, and ${H}_{{en}}$ denote the electron Hamiltonian, nuclear spin Hamiltonian, and the electron-nuclear hyperfine interaction (HFI), respectively.

The electron Hamiltonian, in the presence of an external magnetic field ***B***, includes two terms electron spin–spin interaction and electron Zeeman interaction, and can be written as^105^

2 $${H}_{e}=D({S}_{z}^{2}-S(S+1)/3)+E({S}_{x}^{2}-{S}_{y}^{2})+{g}_{e}{\mu }_{B}{\boldsymbol{BS}}$$

where $D$ and $E$ are the zero-field splitting (ZFS) parameters. ${\boldsymbol{S}}$ represents the total electron spin, with a value of 1 corresponding to a triplet state. The Landé factor, denoted as $g$, and the Bohr magneton, represented by ${\mu }_{B}$, are constants. Additionally, *S*_*x,y,z*_ are the spin-1 operators. ${\gamma }_{e}={g}_{e}{\mu }_{B}$ is the gyromagnetic ratio, which is 28 MHz/mT for the boron vacancy^145^.

The nuclear spin Hamiltonian includes nuclear Zeeman splitting and nuclear-spin quadrupole interaction, and the Hamiltonian can be written as^123^:

3 $${H}_{n}=Q({I}_{z}^{2}-I(I+1)/3)-{g}_{n}{\mu }_{n}{\boldsymbol{BI}}$$

Here $Q$ is the quadrupole coupling constant, $I$ and ${I}_{z}$ are nuclear operators, ${\mu }_{n}$ is the nuclear magneton, the gyromagnetic ratio of ^14^N nuclear spin ${\gamma }_{n}={g}_{n}{\mu }_{n}$ is 3.076 MHz/T^146^.

The hyperfine interaction results from the interaction between the electron spin and the nuclear spin can be described by the following Hamiltonian^123^:

4 $${H}_{{en}}={\boldsymbol{SAI}}$$

Here, ${\boldsymbol{A}}$ is the HFI tensor, ${\boldsymbol{I}}$ is the nuclear spin operator, and ${\boldsymbol{S}}$ is the electron spin operator. The hyperfine coupling constant, resulting from the interaction with ^14^N nuclei (with *I* = 1) in the first coordination shell, has been determined experimentally to be *A* = 47 MHz^105^.

According to Eq. (2), the presence of an external static magnetic field, denoted as *B*, leads to a splitting of the frequencies ν_1_ and ν_2_, as a consequence of the Zeeman effect. This splitting can be described by the equation^105^:

5 $${v}_{1,2}={v}_{0}\pm \sqrt{{E}^{2}+{\left({g}_{e}{\mu }_{B}B\right)}^{2}}/h$$

In this equation, ${\nu }_{0}$, the zero-field splitting frequency, is equal to ${v}_{0}=D/h$, which has a value of 3.480 GHz for the boron vacancy. The zero-field splitting parameter $E$, when divided by *h*, it is 50 MHz^105^. The Landé $g$-factor is 2.

**Fig. 5:**
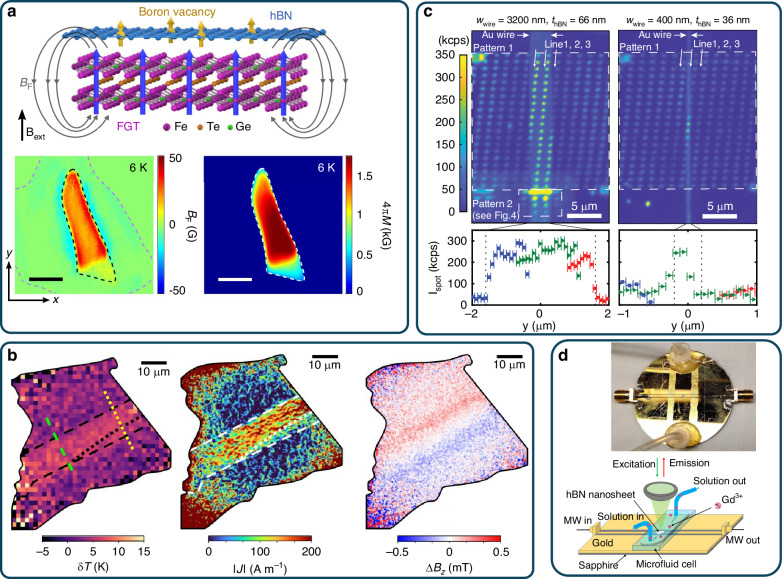
Quantum sensing applications with 2D spin defects. **a** Quantum sensing of nearby stray fields B_F_ produced by generated from Fe_3_GeTe_2_ (FGT)^148^. Two-dimensional maps show the static stray field *B*_F_ and the reconstructed magnetization 4 *M* of a suspended FGT slice, recorded at 6 K with a 142 G perpendicular magnetic field (*B*_ext_). **b** Visualization of Joule heating and current density, stray magnetic fields in a graphene-based device using spin defects in hBN^147^. **c** PL intensity distribution of a sensor array on the hBN flake^168^. **d** An image and a schematic of a microfluidic channel integrated into a gold stripline microwave waveguide for quantum sensing in solution^229^. Panel **a**, **b** are reproduced with permission from refs. ^147,148^, respectively, Copyright by Springer Nature. Panel **c** is reproduced with permission from ref. ^168^, Copyright by American Institute of Physics. Panel **c** is reproduced with permission from ref. ^229^, Copyright by American Chemical Society

So far, several groups have applied ${{\rm{V}}}_{B}^{-}$ center for magnetic field imaging applications. In 2022, Healey et al. demonstrated the visualization of stray magnetic fields in a van der Waals (vdW) ferromagnet, CrTe_2_ (Fig. 5b)^147^. The observed pattern indicated in-plane magnetization, with an amplitude reaching ±1.5 mT, which aligns with a spontaneous magnetization of *M*_*S*_ ~50 kAm^−1^. They achieved time-resolved, simultaneous temperature and magnetic imaging near the Curie temperature of the vdW ferromagnet. Additionally, they mapped charge currents and Joule heating in an operational graphene device. Huang and colleagues studied the vdW ferromagnet Fe_3_GeTe_2_ (FGT) using spin defects within hBN (Fig. 5a)^148^. By employing spin defects in hBN, they visualized magnetic phase transitions and spin fluctuations in the prototype vdW ferromagnet FGT at the nanoscale. They observed a peak in fluctuation magnitude close to the Curie temperature, which is consistent with the anticipated ferromagnetic phase transition. This was achieved through the application of wide-field magnetometry with an ensemble of spin center, which provided spatial resolution only constrained by the fundamental limit of optical diffraction. Kumar also reported on quantitative magnetic imaging using boron vacancies spin defects^149^. The research achieved a sensitivity of approximately 100 μT/√Hz and microscale spatial resolution by diffraction limits. Thin films of hexagonal boron nitride with ${{\rm{V}}}_{B}^{-}$ centers were created through neutron irradiation. These films were then used to capture magnetic images of chromium telluride (CrTe_2_) 2D ferromagnet. It is worth noting that the magnetic sensors based on hBN are highly flexible and compatible with 2D materials that can be placed near target samples. These sensing units are expected to have a significant impact on 2D materials research by providing a straightforward method to in situ study the physics of van der Waals heterostructures.

Sensitivity is a pivotal parameter for a sensor, and in the realm of quantum color center magnetometry, continuous-wave optical detection of magnetic resonance (CW ODMR) has gained widespread adoption due to its simplicity. The sensitivity of a CW ODMR magnetometry is affected by the photon detection rate ($R$), ODMR contrast ($C$), and spectral line width ($\Delta {\rm{\nu }}$), and can be defined as shot-noise-limited sensitivity^146,150^

6 $${\eta }_{{CW}-{ODMR}}=\frac{4}{3\sqrt{3}}\frac{h}{{g}_{e}{\mu }_{B}}\frac{\varDelta \nu }{C\sqrt{R}}$$

The rate of photons can be well estimated based on the power-dependent photon counted intensity. In the case of boron vacancies, the photon count of the ensemble is approximately 10^5^ counts per second. The ODMR contrast is found to be roughly 1%, while the linewidth of the ODMR signal is ~250 MHz^147^. Intriguingly, for the carbon-related defects, the linewidth (Δν) is measured at 10 MHz, the contrast is 30% in recent work^151^. And the brightness $R$ is quantified at 10^5^ events per second for the single defect. This estimated sensitivity is similar to that of the well-established nitrogen-vacancy centers, suggesting their high potential for sensing.

The intrinsic linewidth limit, $\Delta \nu$, is governed by the inverse of the inhomogeneous dephasing time of the defect electron spin ${T}_{2}^{* }$, i.e., $\Delta \nu \sim 1/{T}_{2}^{* }$^20^. In the context of pulsed ODMR, where spin manipulation, spin readout and phase accumulation are separated in time. Microwave/Radio-frequency pulses are applied when the polarization laser is off to minimize the spectral broadening caused by optical and microwave excitation powers. When the pulse sequence is optimized, the spectral linewidth is predominantly dictated by the intrinsic character of the spin transitions and the broadening associated with the duration of the microwave pulse. Consequently, the enhanced sensitivity to a static (DC) magnetic field can be expressed as follows ^20^:

7 $${\eta }_{P-{ODMR}} \sim \frac{h}{{g}_{e}{\mu }_{B}}\frac{1}{C\sqrt{R{t}_{c}{T}_{2}^{* }}}$$

Here, ${T}_{2}^{* }$ is the inhomogeneous dephasing time of the defect electron spin, and ${t}_{c}$ is the photon counting time of each pulse.

Spin defects have also been shown to be sensitive to strain in recent studies^152–154^. By observing the shift in ZFS, we can deduce the relative strain by analyzing modifications to the ZFS parameters. Consequently, strain can be quantified through changes in these parameters exhibited by the defects^152^. Lyu and colleagues utilized spatially resolved Raman and ODMR spectroscopies to analyze the complete strain distribution, including in-plane and out-of-plane components, in hBN flakes^153^. They demonstrated that the ODMR measurement of ${{\rm{V}}}_{B}^{-}$ centers allows access to the strain along the out-of-plane direction through the ODMR measurement of the spin m_s_ = ±1 sublevels.

Nano-scale nuclear magnetic resonance (NMR) is a promising field that uses spin defects^67^. Nuclear spins have longer coherence times than electron spins and can be used as auxiliary memory qubits to increase the sensitivity of advanced pulsed sensing protocols. hBN is a good candidate for NMR applications because it has a rich nuclear spin content, unlike diamond, which has very few^116,155–157^. Every atom in hBN possesses a non-zero nuclear spin, making it useful for quantum sensing applications. Gao et al. used the hyperfine interaction (HFI) between nuclear spins and ${{\rm{V}}}_{B}^{-}$ electron spins to optically polarize the nuclear spins in hBN at room temperature^158^, making it possible to implement optically detected nuclear magnetic resonance (ODNMR). Recently, Ru et al. have showcased the utilization of ground-state level anticrossing (GSLAC) in the ${{\rm{V}}}_{B}^{-}$ center to achieve nuclear spin polarization. This technique is notable for its ability to be performed under the gentle conditions of low-power excitation, paving the way for more energy-efficient and less invasive manipulation of nuclear spins^159^. The ODNMR spectra showed significant nuclear-nuclear coupling mediated by electron spins, which is about 10^5^ times stronger than direct nuclear-spin dipolar coupling. This breakthrough has the potential to transform our ability to determine the structures of proteins, chemicals, and viruses^26,51,160^.

## Engineering optically active defects

**Fig. 6:**
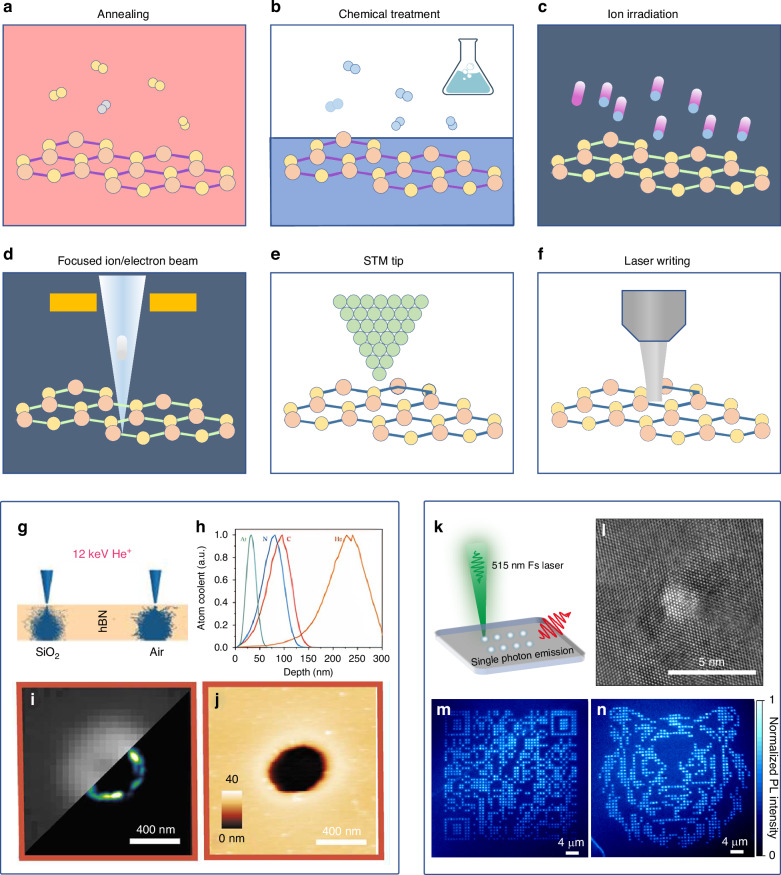
Engineering quantum defects. **a**–**f** Exploration of defect engineering techniques in two-dimensional (2D) materials, encompassing annealing, chemical treatments, ion, and electron irradiation, as well as focused beam, scanning tunneling microscopy (STM) tip, and laser writing methods for manipulating defects in 2D materials. **g**–**j** Focused ion beam write defect in layered materials. Reproduced with permission from ref. ^177^, Copyright by American Chemical Society. **h** Simulation of depth-dependent defect distribution using the stopping-and-range-of-ions-in-matter (SRIM) model, following the implantation of diverse ions such as He, C, N, and Ar. Reproduced with permission from ref. ^175^, Copyright by American Chemical Society. **i** Wield field and single-molecule localization microscopy of the isolated defect site. **j** Atomic force microscopy of the same region in (**i**). Reproduced with permission from ref. ^174^, Copyright by American Chemical Society. **k**–**n** Laser writing color centers in the hBN. Reproduced with permission from ref. ^92^, Copyright by Springer Nature

Following the discovery of optically addressable spin defects in layered, numerous experimental techniques have been developed for engineering these defects on demand. These methods include thermal annealing^95^, plasma^161^, focused ion beam^162^, electron beams (e-beams)^93,99,163,164^, and laser writing^92,112,165–167^, or a combination of different process, as shown in Fig. 6. Overall, there are two main kinds of methods: one involves the large-scale high-density fabrication of point defects (annealing, chemical treatments, ion and electron irradiation, Fig. 6a–c), which can be used for wide-field imaging applications, such as magnetic, electric, and thermal fields^49,149,168,169^; the other one is the targeted fabrication of a controlled single defect (such as focused ion/electron beam, scanning tunneling microscopy tip, and laser writing methods, as shown Fig. 6d–f), which can be applied to high-spatial resolution measurements and may also be used for future nanoscale or single-molecule level scanning imaging^9,170^.

### High-density defects generation in crystal

Thermal annealing and plasma treatment are commonly employed to introduce defects in layered crystals^127^. For example, a two-step process that involves Ar plasma etching followed by annealing in an Ar atmosphere has been demonstrated to be highly effective, resulting in an eightfold increase in emitter concentration in hBN^161^. Chen et al. employed high-temperature annealing in a controlled, oxygen-rich atmosphere^171^, optimizing the process at 1100 °C with a 1000 sccm O_2_ flow rate to achieve a single-photon emitter density of ~0.327 per μm^2^. Lyu et al. have developed an effective technique for triggering single-photon emission through annealing in a carbon-rich ambiance^95^. Utilizing a one-step annealing process in a gas mixture of argon, methane, and hydrogen (Ar:CH_4_:H_2_ = 15:5:1), they significantly enhance the density of single-photon emitters.

Irradiation, which involves the use of neutrons, ions, and electrons, is an important method for inducing high-density defects in materials. For example, the color of hBN changes to pink after neutron irradiation, as reported by Toledo et al.^119^. This color change is the result of the creation of two absorption bands in the bandgap. The first is a broad UV absorption band located near the bandgap, and the second is a broad visible absorption band located at ~490 nm. Together, these absorption bands cause the pink color observed in neutron-irradiated powders. The intensity of both absorption bands increases with the dose of irradiation, indicating the formation of color centers, which are point defects that create additional energy levels within the bandgap of hBN. However, these irradiation-induced pink colorations can be eliminated through thermal treatments conducted at temperatures ranging from 600 to 800 °C. Tran et al. performed electron beam irradiation experiments in a scanning electron microscope within a low-vacuum chamber containing water at 8 Pa^93^. They observed and confirmed the presence of single photon emitters.

### High-precision quantum defect generation

Thermal annealing, plasma treatment, and irradiation with particles are effective methods to create quantum emitters in layered materials. However, achieving the controlled generation of single spin defects remains a challenge and is crucial for high-resolution quantum sensing. Multiple attempts with focused ion implantation^172–177^, electrons^99,164^, and lasers^92,112,165,167,178,179^, have been made over the years to achieve the controlled generation of the single quantum defect.

The use of focused ion beams (FIB) for generating boron vacancy (${{\rm{V}}}_{B}^{-}$) defects has gained prominence. Multiple ion beam options, including nitrogen^106,120^, xenon, argon, helium^175,180^, neon^181^, gallium^176^, and carbon, are available. Kianinia et al. conducted an analysis of the depth of defect creation^106^, revealing that xenon ions are more effective at creating vacancies at a shallower depth, peaking at ~15 nm, compared to argon and nitrogen ions, which peak at depths of around 25 and 60 nm, respectively. Guo et al., on the other hand, utilized argon, nitrogen, helium, and carbon ions to create boron vacancy defects, observing ODMR results^175^. It is noted that the helium ion have much larger creation depth of around 250 nm. Glushkov et al. observed the local amorphization of hBN upon ion beam irradiation^174^. These studies reveal that ions interact not only with the thin hBN flakes but also with the substrate on which they are supported. Additionally, exposure to water causes the amorphized hBN to undergo a transition in structure and optical properties between two defect types with distinct emission characteristics. Sasaki et al. have successfully created a ${{\rm{V}}}_{B}^{-}$ sensor array that measures 20 × 20 micrometers on a hBN flake using helium ion microscope (HIM) irradiation (Fig. 5c)^168^. By utilizing this technology, they were able to demonstrate magnetic field imaging with a nano-array of V_B_ quantum sensors. Each sensor is less than 100 nm thick and has a size of (100 nm)² in hBN. Although FIB is a highly desirable technique to generate optically active defects, most studies have focused on ensembles of emitters rather than single defects at single sites. In the context of two-dimensional (2D) systems, defect generation may be primarily influenced by backscattered ions and sputtered substrate atoms, rather than by direct ion impact^172^. Additionally, the extent of damage in 2D materials is significantly influenced by the presence or absence of a substrate. Consequently, the induction of a solitary spin defect remains a formidable challenge, necessitating precise control over ionic species and processing parameters.

The direct laser writing method has emerged as a means to create point defects in wide-bandgap materials^112,165,178,182^. Gao et al. (2021) showed that femtosecond laser irradiation can generate optically addressable spin ensembles in hBN^112^, displaying a promising ODMR contrast at room temperature. The appeal of laser writing lies in its ease of use and scalability. Subsequently, Gan et al. (2022) produced large-scale single photon emitter arrays from 3.0 μm defect patterns with a 43% yield^166^. However, the sizes of laser-processed materials are typically in the range of micrometers, which is much larger than the size of point defects. As a result, they are not suitable for sensing applications that require high spatial resolution. Additionally, the color centers are randomly distributed around the 3-micron holes/voids. More recently, Xiao et al. developed a new method that enhances laser writing resolution to sub-5-nm spatial resolution by using a threshold tracing and lock-in method^92^. This allowed for the deterministic creation of single photon emitters in regular arrays with 100% yield and high positional accuracy. Despite this breakthrough, the defects exhibit a broad range of emission wavelengths in the visible spectrum, and further validation is required on their spin properties. Wong et al. also demonstrated that individual native defects in bulk hBN can be identified and altered with the aid of a scanning tunneling microscope^183^. However, it should be noted that this method may only be effective for manipulating intrinsic defects.

## Challenges of quantum sensing with 2D spin defects

As discussed above, quantum sensing sensitivity relies on the number of detected photons (*R*) and the spin coherence time (${T}_{2}$)^14^. In a simplified model, the spin-projection-limited sensitivity of an ensemble can be represented as proportional to$\,\eta \propto 1{\rm{ / }}\sqrt{n{T}_{2}}$^17,124^. To improve the sensitivity of a magnetometer, a useful approach is to utilize a large number of sensing spins. This method takes advantage of the high density of spins achievable in a solid-state system. The reason for this improvement is that the collected PL signal is amplified by the number $N$, thus improving the shot-noise limited magnetic field sensitivity by a factor of $1/\sqrt{N}$^33^. However, at high spin densities, the presence of paramagnetic impurities, as well as interactions between spin centers, can potentially hinder the sensitivity of the magnetometer. Whille for nanoscale resolution sensing, the unparalleled sensitivity offered by single spin defects remains indispensable.

For optically active spin sensors applications, especillay for magnetometer with nanoscale resolutionthe, the spin defects ${{\rm{V}}}_{B}^{-}$, however, face certain challenges in improving the sensitivity when compared to the more widely studied nitrogen-vacancy (NV) centers in diamond:

### Low quantum efficiency

The quantum efficiency of the ${{\rm{V}}}_{B}^{-}$ defects is ~0.03% (70% for the NV^-^ center)^108,124,184^. The spectra of the ${{\rm{V}}}_{B}^{-}$ defects is broad, peaking around 820 nm, and lacks a sharp, distinct zero-phonon line (ZPL). Calculations suggest that the ZPL is forbidden, meaning all emitted intensity is associated with vibronic origins^184^. The defect’s triplet to singlet manifolds undergoes rapid intersystem crossing, which leads to a very short excited-state lifetime and low quantum yield. Consequently, enhancing the rate of photon emission ${{\rm{V}}}_{B}^{-}$ defects and the efficiency of photon collection emerges as a critical objective for the advancement of quantum sensing applications. In hBN, many color centers with high brightness have been reported, and searching for color centers with good spin properties is also an important task at the moment.

### Short coherence time

In hBN, the spin coherence time of the ${{\rm{V}}}_{B}^{-}$ is relatively short, which is a drawback for quantum sensitivity. According to ref. ^114^, the coherence time is about 82 ns in neutron-irradiated hBN samples that have not been isotopically purified. In 2023, Ramsay and colleagues studied the spin echo coherence time at room temperature of boron vacancy ensembles in hBN^145^. They discovered that the coherence time is limited to less than 100 ns due to interactions between electrons and nuclei at magnetic fields weaker than 100 mT. However, they were able to temporarily extend the coherence time to ~4 μs by utilizing a strong continuous microwave drive with modulation, which stabilized Rabi oscillations and approached the 10 μs lifetime of the electron spin in their sample. Nevertheless, this extension is only a fraction of the millisecond-scale coherence times observed in diamond^14^, where the ${{\rm{V}}}_{B}^{-}$ coherence times are typically in the tens microseconds range^114,124,145,185–187^.

Despite the current gap in quantum sensing performance between ${{\rm{V}}}_{B}^{-}$ and diamond NV center in diamond, the wide variety of two-dimensional materials and their defects still offer promising opportunities^143,154,188–191^. In particular, hBN, is a promising material that has been widely used as an insulator substrate or encapsulating material^192^, which is indispensable in nanoelectronics and nanophotonics. The van der Waals nature of hBN also allows it to be seamlessly integrated with other 2D materials or substrates^90^. Experiments have demonstrated the potential for miniaturizing and integrating these sensors into 2D heterostructures, paving the way for the development of nanoscale spin sensing techniques. These techniques will be crucial for exploring emergent phenomena in low-dimensional quantum materials and devices, such as superconductivity^193^, ferroelectrics^194,195^, and ferromagnets in 2D materials^196^. Additionally, the thinness of hBN, which can be reduced to a single atomic layer, allows it to approach the sample being measured to the physical limits of proximity.

To facilitate practical applications of quantum sensing utilizing 2D spin defects, there are several aspects worth exploring further:

### Screen spin defects with intrinsic properties for quantum sensing

**Fig. 7:**
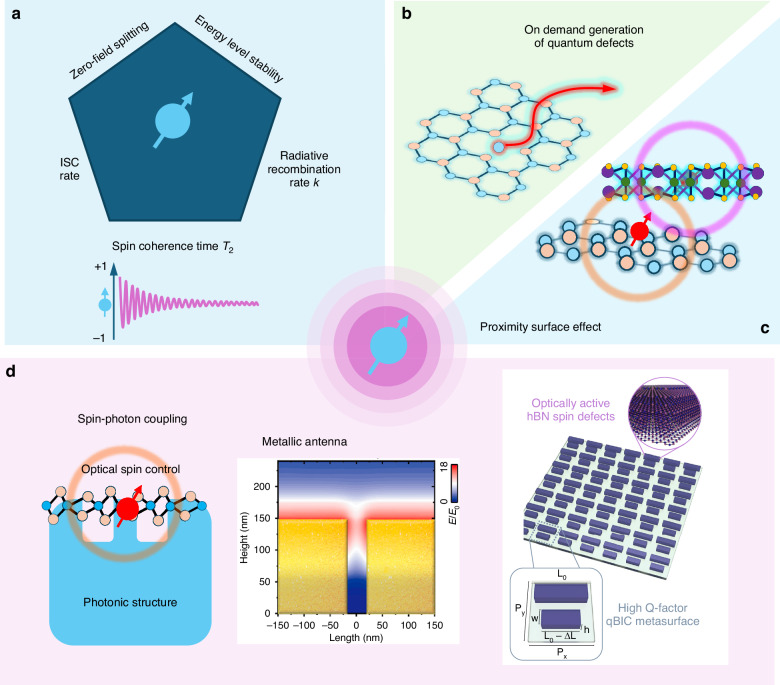
Considerations and approaches for enhancing quantum sensing. **a** Evaluation of spin defects for quantum sensing requires assessment of parameters including spin coherence, radiative recombination, intersystem crossing rates, energy level stability, and zero-field splitting. **b** The controlled generation of spin defects is critical and essential for the functionality of quantum sensing applications. **c** The proximity and surface effects significantly influence the sensing performance of two-dimensional spin defects. **d** The interaction between the spin and photonic structures may be leveraged to manipulate and enhance spin performance in quantum sensing^218,219^. Panel **d** is reproduced with permission from refs. ^218,219^, Copyright by American Chemical Society and Springer Nature, respectively

For quantum sensing applications, a spin defect with certain intrinsic properties is promising, as shown in Fig. 7a. These features include efficient radiative recombination, large intersystem crossing (ISC) rates, and long spin coherence and relaxation times, deep defect levels, sizable zero-field splitting, and so on^140^. Efficient radiative recombination results in a high quantum yield, which means a larger number of detectable photons and higher sensitivity. ISC is essential for optical spin initialization and readout, as it enables spin-dependent population differences and modifies PL count rates between spin states^101^. A high ISC rate may enhance spin polarization and ODMR contrast, making efficient spin state control possible^197^. The spin coherence time and relaxation time (T_1_) are critical parameters that determine the sensor’s performance. A longer T_2_ enabling the detection of more subtle signals and preserving sensitivity during prolonged observations^198^. A greater T_1_ indicates reduced energy dissipation to the environment, which is beneficial for the accurate initialization and retrieval of spin states, as well as for measurements of higher fidelity^101^. Moreover, it should also have deep defect levels, such that they are spatially confined and energetically isolated from direct interactions with delocalized charge carriers^140^. A large zero-field splitting is also imperative for the detection of magnetic field angle variations^199^.

### On demand generation of quantum defects

Generating quantum defects is a challenging task, especially when it comes to creating them on demand (Fig. 7b)^78,200^. Most 2D materials require strict growth conditions to produce high-purity crystals. For example, high purity hBN growth necessitates high temperatures and pressures^201^. While controlling defect formation during growth holds promise for the production of large-area spin defects ensemble, current methodologies primarily rely on post-growth physical and chemical treatments, such as ion implantation. These treatments can easily lead to amorphization of the crystal, introduce disordered defects, and create unwanted defects^174,202^. These issues adversely affect the spin properties of the target spin defects, such as shortening the spin coherence time and reducing quantum yield. To date, no technique has emerged that can reliably generate the desired quantum defects in 2D semiconductors on demand without compromising lattice integrity or precision positioning.

For wide-field quantum sensing and imaging, it will be crucial to generate a large, homogeneous density of spin defects ensemble across a wide area^203^. Ion irradiation, among other methods, will need to be refined to minimize lattice damage and to ensure the production of spin defects with a consistent density and type. To achieve nanoscale magnetic field image mapping with an individual electronic spin, or spin manipulation with coupling nanostructures, precise positioning of defect formation is necessary. Laser processing has been successful in creating near-atomic-sized color centers in hBN^92^. However, the type of defect formed is not controllable. To create specific spin defects, laser-induced nanoscale doping using external atoms such as carbon may be a promising approach. Scanning tunneling microscopy (STM) is a technique that allows manipulating and characterizing the defect at the surface of crystals. It has been recently used to manipulate individual atoms in 2D materials^78,144^. This could potentially lead to the creation of ideal single spin defect. These advancements have significant implications for understanding quantum defects and their potential spin applications.

### Proximity and surface effect on quantum sensing

Recent ODMR experiments have shown that spin defects in hBNs can be utilized for magnetic sensing. These experiments, however, employed hBN flakes that were several tens of nanometers in thickness, significantly thicker than what is considered the 2D limit. This raises the question of whether spin defects maintain their spin-dependent optical response when present in atomically thin layers since the proximity and surface effect are important (Fig. 7c). This question is crucial for the future development of 2D materials-based quantum sensing foils. Recent work by Durand and colleagues highlights the importance of thickness on the electronic spin properties of defects, which evolve with the hBN thickness^204^. They observed a shortening of the T_1_ time and weaker PL intensity in a few-layer sample compared to a thicker sample. However, it is encouraging to note that the T_1_ time increases by three orders of magnitude in a cryogenic environment. The use of two-dimensional materials may not be as straightforward as assuming that the thinner the materials, the higher the sensing sensitivity. When the thickness decreases, other factors such as charge noise from the substrate also significantly affect the properties of spin defects. Charge fluctuations can cause inhomogeneous broadening of the optical transitions associated with the spin defect. This broadening can degrade the signal-to-noise ratio and the overall performance of quantum sensors based on these defects. This phenomenon is analogous to the decrease of nanodiamond size, which increases the influence of surface defects on the spin coherence time of the color center^205^. Moreover, it is essential to take into account dielectric screening, a fundamental characteristic of 2D materials. Research using electric force microscopy (EFM) has investigated the impact of dielectric screening on extremely thin boron nitride nanosheets with varying thicknesses^206^. These studies indicate that dielectric screening in extremely thin BN depends on the thickness. However, the exact effects of thickness on dielectric screening and spin properties are not fully understood yet. On the other hand, dielectric disorder arises from local fluctuations in environmental permittivity^207^. It introduces a spatial modulation of the Coulomb interaction, which in turn can affect the electronic states and dynamics of the spin defects within the sensor. Understanding and mitigating the effects of dielectric disorder is also crucial for enhancing the sensitivity, coherence, and overall functionality of spin sensors in quantum technologies.

When spin host materials reach atomic thickness, the supporting substrate that holds the 2D materials becomes crucial for their spin properties^208^. The interface between the spin host 2D materials and the substrate can significantly impact the spin relaxation dynamics. The surface dangling bonds of the substrate may interact with the 2D materials and introduce impurities that affect spin relaxation through spin-orbit coupling (SOC) modification^208^. The thermal vibrations of substrate atoms can also cause additional spin-phonon scattering by interacting with the spins of the materials. Recent reports show that ferroelectric order emerged at the interface between two naturally grown hexagonal boron nitride flakes^209^, producing a ferroelectric effect that can induce doping in a monolayer semiconductor^210^. The interface makes the spin properties more complex and difficult to predict, therefore requiring careful investigations to unveil its distinct properties.

### Integration of spin defect with photonic structures for novel function and higher emission rate

Recent advances in quantum computing have made significant strides in spin qubit initialization and readout using photonic cavities. For instance, Carter et al. demonstrated quantum control over a spin qubit in a photonic crystal cavity, showcasing the ability to alter spin states using light^211^. Similarly, Yale et al. introduced an all-optical method for spin control in solid-state systems that uses coherent dark states for spin initialization, readout, and manipulation^212^. These works have tremendous implications for quantum sensing, as they have the potential to enhance the functionality of spin defects when used in conjunction with photonic structures.

It is possible to create new photonic structures on a substrate that can detect and manipulate spin states using optical means (Fig. 7d). These structures can improve the performance of spin sensing while keeping the spin very close to the sample. For example, the integration of photonic crystals, microcavities, plasmonic heterostructures, or other optical elements on the surface of a two-dimensional material has been attempted^98,213–217^, which may help confine and control photons locally, thereby enhancing the interaction between optics and spin electronics. This not only improves the efficient reading of spin qubits but also contributes to the sensitivity of sensing.

In a recent study, researchers investigated the potential of using metallic nanotrenches to boost the emission of spin defects in hBN, which could enhance its quantum sensing capabilities^218^. Additionally, the study demonstrated the possibility of incorporating plasmonic heterostructures into coplanar waveguide (CPW) electrodes, resulting in an increase in DC sensitivity up to ∼6 × 10^–5^ T/Hz^1/2^. Sortino and colleagues have used quasi-bound states in the continuum (qBICs) to create high-Q factor cavities and generate localized and strongly enhanced electromagnetic fields^219^. The coupling to these cavities has resulted in a significant spectral narrowing of the defect emissions, with a full width at half maximum (FWHM) of less than 4 nm. The qBIC-driven PL enhancement is achieved by an in-plane field component of the qBIC resonance. The ODMR measurements reveal that the hBN metasurfaces improve spin-readout efficiency and narrowband PL filtering. As layered materials can be easily integrated with photonic structures, combining spin-photon functionalities into a unified chip shows high potential for enhanced quantum sensing.

## Summary and outlook

In recent years, significant advancements have been made in the field of 2D spin defects. Ongoing optimization efforts, including isotope engineering and dynamical decoupling protocols^118,120,124,146,220^, have successfully enhanced sensitivity and extended the coherence time (T_2_) of 2D spin sensors. One of the primary advantages of 2D spin defects is their facile integration and the achievable thinness in the 2D limit, which is impossible for conventional 3D bulk materials. Moreover, beyond hBN, the emergence of new 2D materials with large bandgap characteristics, such as transition-metal oxides (TMOs) like the 2H phase MoO_2_^221^, octahedral α-MoO_3_^222^, has expanded the range of options for hosting 2D spin defects. These novel materials offer numerous additional platforms for the exploration and application of 2D spin defects.

**Fig. 8:**
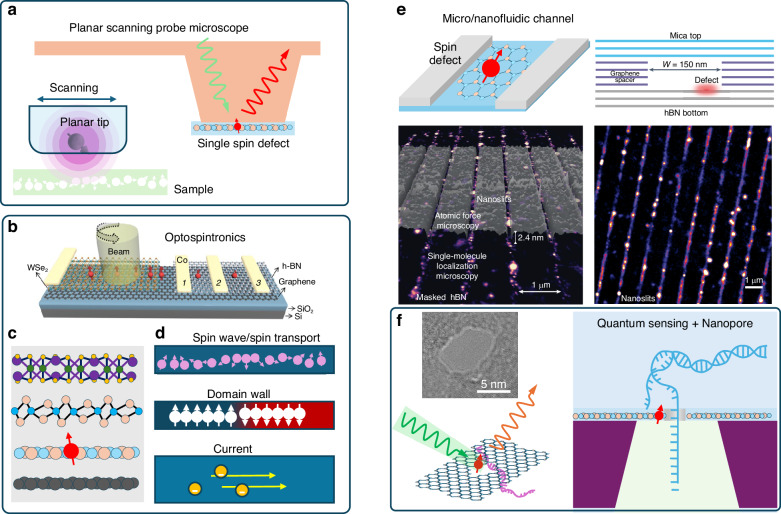
Potential application of 2D spin defects. **a** Schematic of a planar scanning probe microscope, in which the probe uses the spin center within 2D materials. **b** An optospintronic device is constructed from a heterostructure composed of a monolayer of WSe_2_, a monolayer of graphene, and hBN deposited on a conventional SiO_2_ substrate. With reproduce permission from^233^, Copyright by American Chemical Society. **c** Different types of 2D materials. **d** The spin defects in 2D materials can be used to probe various phenomena in 2D nanodevices, like a spin wave, spin transport, ferromagnetic domain, and current flow in devices. **e** Sketch of using 2D spin defect in micro/nanofluidic devices. The plane illustrates a heterostructure nano-slit device. It overlays a super-resolved image showcasing masked ethanol-activated hexagonal boron nitride (hBN) and an atomic force microscopy (AFM) scan mapping the graphene spacers. Additionally, there is a super-resolved image that shows acetonitrile-activated emitters embedded within the nanoslits^230^. Panel **e** is reproduced with permission from ref. ^230^, Copyright by Springer Nature. **f** Schematic of a quantum-enhanced DNA sequencing method by combining nanopore sequencing and quantum sensing using a 2D material membrane fabricated by a femtosecond laser to achieve high chemical resolution. The photo shows a nanopore in an hBN membrane fabricated by a femtosecond laser

Quantum sensors based on 2D materials are currently being used to perform wide-field imaging by utilizing an ensemble of spin defects. However, instead of wide-field imaging, individual spin defects can also be used for high-resolution scanning imaging. Particularly noteworthy is the development by Ernst et al. of a planar scanning probe microscope, that enables imaging with extended planar sensors^223^. This microscope uses a laterally milli-meter-sized bulk diamond sensor based on a combination of far-field optical techniques that measure both tilt and distance between the probe and the sample with sub-mrad and sub-nm precision, respectively. The 2D materials for spin detection can also be directly integrated onto the planar probe (Fig. 8a), thus enabling high-resolution scanning imaging.

The two-dimensional nature of spin defects in 2D materials renders them highly suitable for applications involving nanoscale detection of magnetic and electric fields. For instance, the hBN substrate and encapsulation layers play a crucial role for the engineering of superconductivity properties in graphene systems^224^. They minimize scattering events that might disrupt the coherent superconducting state. Consequently, the spin defects present in the hBN are ideal probes for in situ investigations of the quantum state within two-dimensional (2D) materials. As reported, we can optically access the intrinsic spin transport properties of these materials using spin sensors^225^. This allows for the detection of dynamic fluctuations in the spin density, providing a non-invasive method for studying the quantum state. Furthermore, magnetic imaging techniques can be extended to investigate spintronics^226^, ferrimagnetic textures^227^, as well as ferroelectric domains^228^. These imaging capabilities will provide valuable insights into the spatial distribution and arrangement of magnetic moments within these materials, thereby enhancing our understanding of their fundamental properties and behavior. These properties are particularly valuable in investigating the physical properties of 2D nanodevices, as shown in Fig. 8b.

Similar to the diamond, hBN exhibits biocompatibility and chemical stability, making it highly suitable for biological applications. In a recent study, Gao et al. demonstrated that spin defects in hBN can detect paramagnetic ions in liquids effectively (Fig. 5d)^229^. These spin defects, located near the surface of hBN, exhibit a high contrast in ODMR signals in liquid environments. This property was utilized to detect paramagnetic ions such as Gd^3+^ in water with high sensitivity, reaching ~10^–18^ mol/L via spin relaxation measurements.

Moreover, recent studies have shown that 2D materials can be used to fabricate nanofluidic structures(Fig. 8e), for investigating their quantum optical properties in liquid within heterostructure nano-slit device^230^. Incorporating spin defects into these structures may enable the investigation of the dynamics of liquids confined to the nanoscale, which is valuable for the field of chemistry. The use of 2D materials nanopores for site-specific electronic recognition of DNA-nicks has also been reported^231,232^. It has been observed that spin defects can be generated by laser processing, which are distributed around the periphery of the processed voids. This suggests that these defects could potentially form near nanopores. If laser processing techniques can be meticulously controlled, we could integrate nanopores with optically accessible spin defects to harness quantum sensing techniques for DNA detection. Therefore, we could introduce a novel quantum-enhanced DNA sequencing method that combines nanopore sequencing with quantum sensing, utilizing a 2D material membrane to achieve high chemical resolution (Fig. 8f). In the context of single DNA analysis, we imagine that as DNA molecules translocate through the nanopores, they can be detected by the spin defect in the rim, thereby providing sequence information.

The 2D spin defect has great potential applications in physics and biology. However, there are also many challenges to overcome. Presently, there is no particularly excellent spin defect, and for instance the ${{\rm{V}}}_{B}^{-}$ emission efficiency is low while the spin lifetime is short. Its sensitivity is still much lower than that of the NV centers. Nevertheless, with the advancement of materials technology and defect engineering techniques, 2D spin defects will become increasingly important.
